# Risk of Hepatitis B Virus Reactivation in COVID-19 Patients Receiving Immunosuppressive Treatment: A Prospective Study

**DOI:** 10.3390/jcm13206032

**Published:** 2024-10-10

**Authors:** Nicoleta Mihai, Mihaela Cristina Olariu, Oana-Alexandra Ganea, Aida-Isabela Adamescu, Violeta Molagic, Ștefan Sorin Aramă, Cătălin Tilișcan, Victoria Aramă

**Affiliations:** 1 Faculty of Medicine, University of Medicine and Pharmacy ‘Carol Davila’, No 37, Dionisie Lupu, 020021 Bucharest, Romania; nicoleta.mihai1@drd.umfcd.ro (N.M.); oana-alexandra.ganea@drd.umfcd.ro (O.-A.G.); violeta.molagic@umfcd.ro (V.M.); victoria.arama@umfcd.ro (V.A.); 2 National Institute of Infectious Diseases ‘Matei Bals’, 1 Dr. Calistrat Grozovici, 021105 Bucharest, Romania; aida-isabela.adamescu@drd.umfcd.ro (A.-I.A.); sorin.arama@umfcd.ro (Ș.S.A.); catalin.tiliscan@umfcd.ro (C.T.); 3 Faculty of Dental Medicine, University of Medicine and Pharmacy ‘Carol Davila’, No 37, Dionisie Lupu, 020021 Bucharest, Romania

**Keywords:** SARS-CoV-2, COVID-19, immunosuppressive agents, hepatitis B virus, reactivation

## Abstract

**Objectives:** This study aimed to evaluate the risk of hepatitis B virus reactivation (HBVr) in COVID-19 patients receiving immunosuppressive treatment, which has been insufficiently studied to date. Secondarily, we aimed to evaluate the seroprevalence of HBV infection in COVID-19 patients. **Methods:** We performed HBV screening on all Romanian adults hospitalized in four COVID-19 wards between October 2021 and September 2022. We enrolled patients with positive hepatitis B core antibody (anti-HBc) without protective hepatitis B surface antibody (anti-HBs), HBV treatment, or baseline immunosuppressive conditions, and we conducted a virological follow-up on these patients at 3 months. **Results:** We identified 333/835 (39.9%) anti-HBc-positive patients. Follow-up was performed for 13 patients with positive hepatitis B surface antigen (HBsAg) and 19 HBsAg-negative/anti-HBc-positive patients. Among those who received immunosuppressants, 4/23 (17.4%) patients experienced HBVr: 1 out of 8 (12.5%) HBsAg-positive patients (with 1.99 log increase in HBV DNA level) and 3 out of 15 (20%) HBsAg-negative/anti-HBc-positive patients (with a de novo detectable HBV DNA level). **Conclusions:** Administration of COVID-19 immunosuppressants may result in a significant risk of HBVr in co-infected patients. We recommend performing an HBV triple screen panel (HBsAg, anti-HBs, anti-HBc) for all COVID-19 patients receiving immunosuppressive treatment. HBV prophylaxis may be indicated in certain patients. Larger studies are needed in order to establish appropriate and cost-effective management for these patients.

## 1. Introduction

The SARS-CoV-2 infection has generated one of the most critical and unprecedented public health crises in the modern world, having caused more than 776 million cases and 7 million deaths globally by 18 August 2024 [1]. The main pathophysiological mechanism implicated in the occurrence of the severe forms described in COVID-19 is immune hyperreactivity, i.e., the uncontrolled release of pro-inflammatory cytokines [2]. In an attempt to withstand this “cytokine storm”, treatment protocols for COVID-19 include systemic corticosteroids, interleukins (IL) 1 and 6 receptor antagonists, and Janus kinase inhibitors [3]. However, these drugs pose a risk of hepatitis B virus (HBV) reactivation in patients co-infected with SARS-CoV-2 and HBV.

To date, there are only a few studies [4,5,6,7] with small samples that have followed the occurrence of HBV reactivation (HBVr) in COVID-19 patients receiving immunosuppressive treatment. In addition, the varying design of these studies makes it difficult to draw a conclusion. Thus, there is no consensus regarding the management of HBV-infected patients receiving COVID-19 immunosuppressants. This topic deserves attention all the more, since the majority of the world’s population lives in regions with intermediate or high HBV endemicity [8].

Thus, we aimed to present more data to fill these research gaps by conducting a prospective study to evaluate the risk of HBVr in COVID-19 patients receiving immunosuppressive treatment. In addition, we set out to find a common denominator for all studies related to HBVr in COVID-19 patients in order to have an overview of the results to date and formulate management recommendations. As a secondary objective, we aimed to evaluate the seroprevalence of HBV infection in COVID-19 patients to clarify the importance of assessing the risk of HBVr in these patients.

## 2. Materials and Methods

### 2.1. Study Design and Participants

We performed a prospective observational cohort study in which we included patients co-infected with SARS-CoV-2 and HBV admitted to the National Institute of Infectious Diseases “Matei Bals” (NIIDMB) in Bucharest, Romania.

We requested hepatitis B surface antigen (HBsAg), hepatitis B surface antibody (anti-HBs), hepatitis B core antibody (anti-HBc), hepatitis C virus antibody (anti-HCV), and human immunodeficiency virus (HIV) testing for all Romanian patients aged ≥18 years diagnosed with SARS-CoV-2 infection (by polymerase chain reaction (PCR) and/or rapid antigen test) who were hospitalized in 4 of the NIIDMB wards (III, IV, XI, and XIII) between October 2021 and September 2022.

Patients who had positive HBsAg or negative HBsAg/positive anti-HBc were considered for inclusion in the study. Subsequently, we excluded patients with positive anti-HBs, including borderline values (≥8 IU/mL), those with antiviral treatment for HBV, patients with immunosuppressive diseases (e.g., HIV, leukaemia), as well as those receiving immunosuppressive treatment for conditions other than COVID-19. Patients who met these criteria were asked to participate in the study. Those who accepted enrolment signed an informed consent form. We included both patients who received immunosuppressive treatment for COVID-19 and those without such therapy (the latter represented the control group). 

A blood sample was collected in a tube without anticoagulant from all patients at the time of enrolment. The sample was centrifuged at 3700 revolutions per minute (rpm) for 6 min at 24 °C, and the serum was stored at −20 °C for the subsequent determination of HBV viral load. For patients in whom HBV DNA was undetectable at follow-up, determination of baseline viral load was not considered necessary and was no longer performed.

Patients received the nationally recommended treatment for SARS-CoV-2 infection, depending on the severity of the disease, the availability of drugs, and the decision of the attending physician.

Enrolled patients were called for follow-up (clinical examination and blood collection) 3 months after the last dose of immunosuppressive treatment and, respectively, after discharge if they did not receive such treatment.

The study was approved by the Bioethics Committee of NIIDMB (C11454/24 September 2021).

### 2.2. Data Collection

We entered patient data into two Microsoft Office Excel databases. The first included the age, sex, and the result of viral serological markers of all COVID-19 patients who were screened for HBV as above-mentioned. The second database concerned the data of enrolled patients: demographics, comorbidities, body mass index (BMI), severity of COVID-19, immunosuppressive treatment received during hospitalization, and laboratory data (including those from follow-up). We included the number of lymphocytes (determined using Beckman Coulter DxH Haematology Analyser, Siemens ADVIA 2120i Haematology System—Munchen, Germany, or Celltac G Nihon Kohden Haematology Analyser—Tokio, Japan); prothrombin concentration (determined using Siemens Sysmex CS-2500i Coagulation Analyzer—Kobe, Japan or Instrumentation Laboratory ACL Top 550 Coagulation Analyzer—Bedford, MA, USA); serum level of alanine aminotransferase (ALT), aspartate aminotransferase (AST), gamma-glutamyl transferase (GGT), and total bilirubin (determined using Ortho Clinical Diagnostics VITROS 4600/5.1 FS Chemistry System—Rochester, NY, USA); serological markers for HBV, HCV, and hepatitis D virus (HDV) infections (determined using Ortho Clinical Diagnostics VITROS 3602 Immunodiagnostic System—Rochester, NY, USA); and HBV viral load (determined by real-time quantitative PCR using Roche Cobas 6800 Systems—Bucharest, Romania, with the lower limit of quantification of 10 IU/mL and the lower limit of detection of 3 IU/mL). 

### 2.3. Definitions

The following drugs were considered immunosuppressive treatment: systemic corticosteroids, IL-1 and IL-6 receptor antagonists (anakinra and tocilizumab, respectively), and/or Janus kinase inhibitors (baricitinib), in any dose or duration.

For HBsAg-positive patients, HBVr was defined as (1) ≥2 log (100-fold) increase in HBV DNA level, if previously detectable; or (2) HBV DNA level >100 IU/mL, if previously undetectable. 

For HBsAg-negative/anti-HBc-positive patients, HBVr was defined as (1) detectable HBV DNA, if previously undetectable; or (2) reverse HBsAg seroconversion (reappearance of HBsAg).

The severity of COVID-19 was defined as (1) mild illness, if the patient had symptoms of acute upper respiratory tract infection but no lung involvement; (2) moderate illness, if there was lung involvement without oxygen requirement or with low-flow oxygen requirement (below 6 L/min); (3) severe illness, if there was lung involvement and high-flow oxygen requirement (≥6 L/min); or (4) critical illness, if the patient was admitted to the Intensive Care Unit requiring non-invasive or invasive ventilatory support.

### 2.4. Statistical Analysis

The collected data were analyzed using IBM^®^ SPSS^®^ Statistics, Version 23.0, New York, NY, USA (released 2015). We used the Shapiro–Wilk test to evaluate the distribution of quantitative variables, which proved to be non-Gaussian. Consequently, we presented the median and the interquartile range (IQR) for quantitative variables and the frequencies for nominal and ordinal variables. We used the Mann–Whitney test for bivariate analysis of the variables except for the dichotomous variables, for which we applied the chi-square test (or Fisher’s exact test when the expected frequencies were under 5). A *p* value below 0.05 was required for statistical significance.

## 3. Results

### 3.1. Seroprevalence of HBV Infection in COVID-19 Patients

Between October 2021 and September 2022, 1289 adults were admitted to four COVID-19 wards of NIIDMB. Among them, 1201 patients were screened for HBV. The median age of these patients was 68 years (IQR 54–78), with a range of 18–97 years. More than half of the patients (55%) were female. For 816 (68%) of the patients, the result of all viral markers was available. For the other 32% of patients, some viral markers (especially anti-HBc) were unavailable. We considered positive hepatitis B e-antibody (anti-HBe) and positive hepatitis D virus antibody (anti-HDV) to be equivalent to positive anti-HBc in two patients and one patient, respectively, in whom anti-HBc was not available. Negative anti-HBe was not considered equivalent to negative anti-HBc, given that anti-HBe may disappear over time in patients with resolved infection.

**Figure 1:**
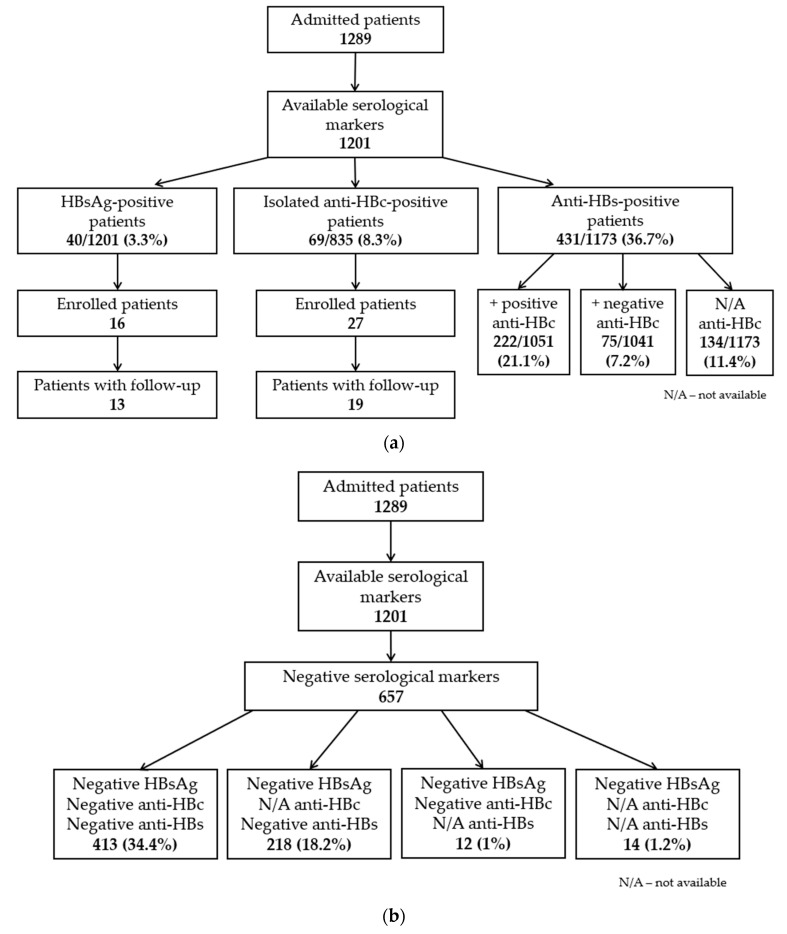
Results of HBV screening in COVID-19 patients: (**a**) Patients with positive serological markers; (**b**) patients with negative serological markers.

We identified 333/835 (39.9%) anti-HBc-positive patients: 40/1201 (3.3%) HBsAg-positive patients, 69/835 (8.3%) isolated anti-HBc-positive patients, 222/1051 (21.1%) anti-HBs/anti-HBc-positive patients, and 2 HBsAg-negative/anti-HBc-positive patients with unknown anti-HBs (Figure 1a). Positive anti-HBc was more frequently found in men (178, 53.5%) than in women (155, 46.5%) (*p* < 0.001). The median age of these patients was 72 years (IQR 64–80), with a range of 26–94 years. A total of 413 patients (34.4%) showed all negative viral markers (Figure 1b). In addition, 50 anti-HCV-positive patients (4.2%) were identified. In total, 18 out of 835 patients (2.2%) were co-exposed to HBV and HCV (they concurrently had positive anti-HBc and anti-HCV).

### 3.2. General Characteristics of Study Participants

A total of 44 patients accepted enrolment in this study and met the inclusion/exclusion criteria. Of these, one patient died and eleven were lost to follow-up. The final cohort included 32 patients: 19 males (59.4%) with a median age of 67 years (IQR 61.5–74.5) and 13 females (40.6%) with a median age of 63 years (IQR 56–69). Most patients (n = 28, 87.5%) had associated comorbidities. The most common was hypertension (n = 20, 62.5%). The Charlson comorbidity index ranged from 1 to 5 (median 3, IQR 2–4). A total of 56% of patients were diagnosed with COVID-19 during the Omicron variant circulation (starting at the end of December 2021). Moderate forms of COVID-19 were predominant. Summary characteristics of study participants are presented in Table 1. More detailed characteristics of each study participant, including the administered treatment and viral loads, are provided in Table 2.

### 3.3. Hepatitis B Virus Status of the Study Participants

#### 3.3.1. HBsAg-Positive Patients

A total of 13 patients were HBsAg-positive. Of these, nine had known chronic HBV infection. The other four patients were newly diagnosed, following the screening performed at admission. One of these patients (no. 12) had concurrently positive anti-HBs (titer 164 IU/mL), and both HBsAg and anti-HBs remained positive at subsequent follow-ups. All HBsAg-positive patients had negative hepatitis B e-antigen (HBeAg), positive anti-HBe, and negative serological markers for HDV (HDVAg and anti-HDV). In addition, one patient was anti-HCV-positive but HCV RNA-negative.

#### 3.3.2. HBsAg-Negative/Anti-HBc-Positive Patients

In total, 19 patients were HBsAg-negative/anti-HBc-positive. All these patients had negative anti-HBs. Among them, one patient (no. 14) was known to have chronic HBV infection (the last medical evaluation 10 years ago). Thus, we documented spontaneous seroclearance of HBsAg. Patient no. 30 was positive for anti-HDV IgG, and patient no. 17 was positive for anti-HCV (HCV RNA was 1,927,058 IU/mL).

A patient (no. 15) who initially had isolated anti-HBc showed positive anti-HBs at follow-up (titer 16 IU/mL). It should be noted that this patient presented severe lymphopenia (lymphocyte count nadir was 450/μL) during hospitalization, which resolved by the time of follow-up. All other patients showed no change in the serological profile of viral markers.

### 3.4. Biochemical and Hematological Parameters of Study Participants

During hospitalization, 19 patients (59.4%) had elevated aminotransferases. For most patients (n = 15, 78.9%), their levels were between one and three times the upper limit of normal (ULN), with only one patient (5.3%) presenting ALT greater than five times the ULN. On follow-up, slightly elevated levels of aminotransferases persisted in five patients (15.6%). In a single patient (no. 17, co-infected with HCV), we detected levels of ALT (6 × ULN) and AST (4 × ULN) higher than during hospitalization. Subsequently, this patient received interferon-free antiviral treatment for HCV infection and achieved a sustained virological response. He did not experience HBVr during therapy.

Elevated levels of GGT were detected in 15/31 patients (48.4%). Most patients (n = 11, 73.3%) showed values between one and three times the ULN. Levels greater than five times the ULN were observed in only one patient. On follow-up, five patients (15.6%) still had slightly elevated GGT. In only one patient, we detected a GGT level greater than three times the ULN. The median of total bilirubin was 0.7 mg/dL (IQR 0.5–0.9), with only two values above the ULN (the highest was 1.6 mg/dL). Biochemical parameters (AST, ALT, and GGT) of each enrolled patient are presented in Table S1.

No patient showed significant alterations in standard coagulation tests. The median of prothrombin concentration was 86.5% (IQR 77.1–91).

Lymphopenia was detected in 24 patients (75%). The median in lymphocyte count nadir was 900/µL (IQR 570–1200). On follow-up, two patients still had mild lymphopenia.

### 3.5. Immunosuppressive COVID-19 Treatment Administered during Hospitalization

The majority of the enrolled patients received corticosteroids (n = 23, 71.9%), mostly intravenous (IV) dexamethasone. Only one patient (no. 18) received a single IV dose (150 mg) of methylprednisolone. Another two patients (no. 6 and no. 23) received oral methylprednisolone at discharge for gradual tapering of corticosteroid therapy. The duration of the treatment as well as the cumulative doses of dexamethasone or equivalent are specified for each patient in Table 2.

Moreover, 12 patients (37.5%) received treatment with tocilizumab, anakinra, and/or baricitinib in combination with corticosteroids (Table 1 and Table 2). 

### 3.6. Patients with HBVr

Patients were reassessed after a median period of 13 weeks (IQR 12–15) from the last dose of immunosuppressive treatment or from discharge if they did not receive such treatment.

According to the criteria we mentioned in the Section 2, 4 out of 23 patients (17.4%) who received immunosuppressive treatment had HBVr: 1 out of 8 HBsAg-positive patients (12.5%) and 3 out of 15 HBsAg-negative/anti-HBc-positive patients (20%) (Table 2). These were the following:

1. Patient no. 8, aged 56, with positive HBsAg and a baseline HBV DNA level of 179 (2.25 log) IU/mL, was treated with IV dexamethasone (8 mg/day for 5 days and then 4 mg/day for 2 days). The patient was initially re-evaluated 1 month after discharge, and at that time, HBV DNA was 1660 (3.22 log) IU/mL. At 3 months, a viral load of 17,378 (4.24 log) IU/mL was detected. FibroMax was also performed, which revealed F1/A0-A1/S3/N2/H0. According to the decision of her attending physician, treatment with entecavir 0.5 mg/day was then initiated.
2. Patient no. 14, aged 75, was known to have chronic HBV infection. However, we found isolated anti-HBc and undetectable HBV DNA at the time of admission. He received a single dose (100 mg) of subcutaneous anakinra, a single dose (400 mg) of IV tocilizumab, and IV dexamethasone (8 mg/day for 3 days; then, 6 mg/day for 5 days, followed by 4 mg/day for 7 days). At follow-up, a detectable viral load was found (HBV DNA < 10 IU/mL).
3. Patient no. 20, aged 63, with isolated anti-HBc and initially undetectable HBV DNA, developed a critical form of COVID-19 requiring admission to the Intensive Care Unit and non-invasive ventilation. During hospitalization, she received a single dose (800 mg) of IV tocilizumab, oral baricitinib (4 mg/day for 3 days), and IV dexamethasone in gradually decreasing doses (initially, 8 mg every 12 h for 1 day; then, 12 mg/day for 3 days; then, 8 mg/day for 7 days, 6 mg/day for 4 days, 4 mg/day for 1 day, and 2 mg/day for 2 days). At follow-up, a detectable viral load was found (HBV DNA < 10 IU/mL).
4. Patient no. 28, aged 80, with isolated anti-HBc and initially undetectable HBV DNA, was treated with IV dexamethasone (6 mg/day for 7 days; then, 4 mg/day for 2 days). At follow-up, detectable HBV viral load (10 IU/mL) was found.

None of the HBVr cases had clinical, serological, or biochemical complications. 

Three of the four patients with HBVr (nos. 8, 20 and 28) were diagnosed with COVID-19 during the Omicron variant circulation.

HBVr did not correlate with sex, age, type of immunosuppressive treatment, severity of COVID-19, or SARS-CoV-2 variant.

## 4. Discussion

### 4.1. Seroprevalence of HBV Infection in COVID-19 Patients

Although the overall prevalence of HBV infection in Europe is low, there are still countries in southern and eastern Europe with intermediate endemicity [9]. In our study, we identified an HBV prevalence (positive anti-HBc) of 39.9% in hospitalized patients with SARS-CoV-2 infection. Positive anti-HBc was found more frequently in men and in patients over 64 years of age. Positive anti-HBc was also identified in younger age groups, starting with the age of 26, despite the implementation of universal childhood vaccination. Although the majority of anti-HBc-positive patients also presented anti-HBs, the degree of protection conferred by them may decrease over time. In addition, it is noteworthy that 8.3% of patients had isolated anti-HBc, while only 3.3% had positive HBsAg. This emphasizes the importance of performing a triple test panel for HBV screening, consisting of HBsAg, anti-HBc, and anti-HBs, as the Centers for Disease Control and Prevention (CDC) also stated in its recent recommendations [10]. Unfortunately, screening is often limited to HBsAg testing. This may result in misidentifying patients at risk of HBVr.

The high HBV exposure in COVID-19 patients identified in this study justifies the concern related to the risk of HBVr in patients receiving immunosuppressants.

### 4.2. Definitions of HBVr

HBVr is broadly defined as a sudden increase in HBV DNA levels or the reappearance of HBsAg in patients with resolved infection [11]. However, to date, there is no consensus in establishing clear limits for HBVr. In Table 3, we summarize the definitions of HBVr proposed by the medical associations dedicated to the management of liver diseases. In our study, we considered the definitions of HBVr that prevailed (see Section 2.3).

### 4.3. Risk of HBVr in Patients Receiving Immunosuppressive Treatment for COVID-19

In our study, we assessed the risk of HBVr in hospitalized patients co-infected with SARS-CoV-2 and HBV who received immunosuppressive treatment. We included both HBsAg-positive and HBsAg-negative/anti-HBc-positive patients for whom we performed a virological follow-up by determining HBV DNA level at baseline and after a median period of 13 weeks from the end of the immunosuppressive treatment. We excluded patients with protective factors for HBVr (those with antiviral treatment for HBV and/or with positive anti-HBs). Of the 32 patients who were followed, 4 had HBVr. However, none of these reactivations were clinically manifest. Only in one case was it necessary to initiate antiviral treatment with entecavir. All patients with HBVr received at least one immunosuppressive treatment (tocilizumab, anakinra, baricitinib and/or systemic corticosteroids). Given that SARS-CoV-2 may itself represent a trigger for HBVr [15,16], we also included a control group (patients with SARS-CoV-2 infection without immunosuppressive treatment). Still, we have not documented any case of HBVr among them.

To date, there are few studies that have evaluated the occurrence of HBVr in patients with SARS-CoV-2 infection receiving immunosuppressive treatment. Additionally, these studies included only a small number of patients without protective factors for HBVr.

A retrospective study conducted in 2020 by Liu et al. included 20 patients with COVID-19 and chronic HBV infection (positive HBsAg) who were monitored virologically during hospitalization. Only five of them received immunosuppressive treatment (methylprednisolone). The authors considered as HBVr a 2log increase in HBV DNA level in patients with previously detectable viremia and the reappearance of HBV DNA in patients with previously undetectable viremia. According to these criteria, three patients with HBVr were identified. Of these, only two received methylprednisolone for 4 days (dose not specified). The third, who had only inhaled interferon alfa-1b, had a small increase in HBV DNA level (initially undetectable, 20 IU/mL at follow-up) [4]. This last situation is not considered HBVr by most guidelines [12,13,14].

A prospective study conducted by Tajez et al. in 2020 included 61 HBsAg-negative/anti-HBc-positive patients with severe COVID-19, of which 38 patients received entecavir prophylaxis. Among the 23 patients without antiviral prophylaxis, 17 patients had positive anti-HBs. Thus, there were only six patients with isolated anti-HBc and without prophylactic treatment. Viral serological markers and HBV DNA level were determined for all these patients 1–2 months after the last dose of immunosuppressive treatment. No case of reverse seroconversion was identified, though two of the six patients with isolated anti-HBc had a detectable HBV DNA level (<10 IU/mL). However, it should be mentioned that no baseline viral load was performed. Both patients received treatment with IL-6 receptor antagonists (siltuximab and tocilizumab, respectively). One of them additionally received methylprednisolone (250 mg/day for 3 days), followed by treatment with prednisone (0.5 mg/kg for a duration that was not specified but was inferred to be at least 1 month) [5].

Another prospective study, conducted by Camarero et al. in the same year, followed 11 patients with SARS-CoV-2 infection and positive HBsAg. The HBV DNA level was determined during hospitalization for only four of the patients who received immunosuppressive treatment. Of these, one received entecavir. Among those without prophylaxis, one patient, who received methylprednisolone (250 mg/day for 3 days), had a 1.48 log increase in HBV DNA level compared to a previous viral load. This was considered HBVr [6].

A recently published study conducted by Foo et al. in 2021–2022 included 54 HBsAg-negative/anti-HBc-positive patients who received tocilizumab or baricitinib for SARS-CoV-2 infection. Of these, only four patients had isolated anti-HBc. The others had protective anti-HBs. All patients were monitored for 3 months, but only serological tests were performed. No reverse seroconversion was identified [7].

Another study, conducted by Yang et al. in 2022, aimed to assess the risk of death/critical COVID-19 in patients with different stages of HBV infection. They also followed six patients with HBVr (defined by the reappearance of HBsAg) but did not report details of these cases (e.g., whether they received immunosuppressive treatment for COVID-19) [17].

Studies related to the risk of HBVr in patients with SARS-CoV-2 infection receiving anakinra are almost nonexistent. Only Mastroianni et al. included 30 patients who received anakinra. However, all these patients were anti-HBs-positive/anti-HBc-positive and received tenofovir prophylaxis [18]. Thus, it is not surprising that no case of HBVr has been identified. 

In addition to these studies, several case reports of HBVr in COVID-19 patients who received corticosteroid therapy [19,20,21] or tocilizumab [22] have been published.

As each previously mentioned study considered different definitions for HBVr and had different inclusion criteria, it was necessary to find a common denominator. Therefore, in Table 4, we presented the number of patients with HBV–SARS-CoV-2 co-infection and immunosuppressive treatment, without protective factors for HBVr, who were followed virologically (by HBV DNA) and experienced HBVr according to the definitions proposed by the medical associations dedicated to the management of liver diseases (see Table 3 as well). We also included the results of the study we reported in this article.

### 4.4. Risk of HBVr in Patients Receiving Immunosuppressive Treatment for Non-COVID-19 Diseases

Most of the data related to the risk of HBVr associated with immunosuppressive drugs used for COVID-19 treatment (corticosteroids, tocilizumab, baricitinib) come from previous studies conducted with patients with other diseases.

Regarding corticosteroids, a study published by Wong et al. in 2019 reported an increase in HBV DNA level of at least 1 log in 303 of 678 HBsAg-positive patients (44.7%) monitored for 1 year, most receiving a short course of corticosteroid treatment (less than 7 days) [23]. In another study, published in 2020, Wong et al. followed 970 patients with isolated anti-HBc who received corticosteroid therapy, showing an annual risk of reverse HBsAg seroconversion of 1.8%, regardless of dose or duration of treatment [24]. It is worth mentioning that, in particular, corticosteroids produce a risk of HBVr due to the presence of a steroid-responsive area in the viral genome [9].

Data on tocilizumab therapy come mainly from patients with rheumatoid arthritis. Although a small number of HBsAg-positive patients without antiviral prophylaxis were included (a total of 10 patients), the three studies performed to date show a high risk of HBVr (60%) [25,26,27]. In addition, several reports of severe cases, some fatal, in HBsAg-positive patients receiving tocilizumab have been published [28,29]. A systematic review conducted by Campbell et al. in 2021 identified eight observational cohort studies that included 192 HBsAg-negative/anti-HBc-positive patients who received tocilizumab but no antiviral prophylaxis. In these patients, the pooled rate of HBVr was 2.6% (5/192) [30,31].

Regarding baricitinib, there is a study published by Harigai et al. in 2020 which included 215 HBsAg-negative/anti-HBc-positive patients with rheumatoid arthritis (201 with positive anti-HBs; 14 with isolated anti-HBc and undetectable HBV DNA at baseline). At follow-up, 32 patients (14.9%) had a detectable HBV DNA level. Seven of these patients received concomitant corticosteroids [32]. To the best of our knowledge, there is no study following HBsAg-positive patients on baricitinib.

To date, no cases of HBVr have been reported in patients receiving anakinra [33]. However, there are almost no studies that have specifically followed this adverse event. We identified a single study that included three HBsAg-negative/anti-HBc-positive patients with rheumatoid arthritis who received anakinra and did not experience HBVr [34].

### 4.5. Management of HBV–SARS-CoV-2 Co-Infected Patients Receiving Immunosuppressive Treatment

To date, there are no recommendations regarding the management of patients co-infected with SARS-CoV-2 and HBV who receive immunosuppressive treatment issued by a medical association dedicated to liver diseases. Thus, currently, the decision to monitor the patient or to administer antiviral prophylaxis depends on the internal protocol of the institution where the patient is admitted and/or on the decision of the attending physician.

From some clinicians’ point of view, it is not necessary to administer HBV prophylaxis in COVID-19 patients receiving immunosuppressants. Regarding corticosteroid therapy, the argument made is the short duration of treatment, generally less than 4 weeks (time limit beyond which prophylaxis would have been recommended) [11]. As for the other immunosuppressive treatments used for SARS-CoV-2 infection (tocilizumab, anakinra, baricitinib), viral prophylaxis is not taken into account, as they are not on the list of drugs associated with high or moderate risk of HBVr [13]. These are also the reasons why the patients included in this study did not receive HBV prophylaxis (according to the decision of their attending physicians).

On the other hand, given that high doses of corticosteroids are used in the treatment of COVID-19, even if for a short period of time, some authors recommend the administration of HBV prophylaxis in all HBsAg-positive patients, as well as in those HBsAg-negative/anti-HBc-positive with a detectable viral load [31,35,36]. HBV prophylaxis could also be considered in HBsAg-negative/anti-HBc-positive patients with an undetectable viral load [35,37]. The same recommendations are made for other immunosuppressive drugs such as tocilizumab and baricitinib [31,36]. However, no recommendations have been made for COVID-19 patients receiving anakinra, as this drug is less commonly used. 

**Figure 2:**
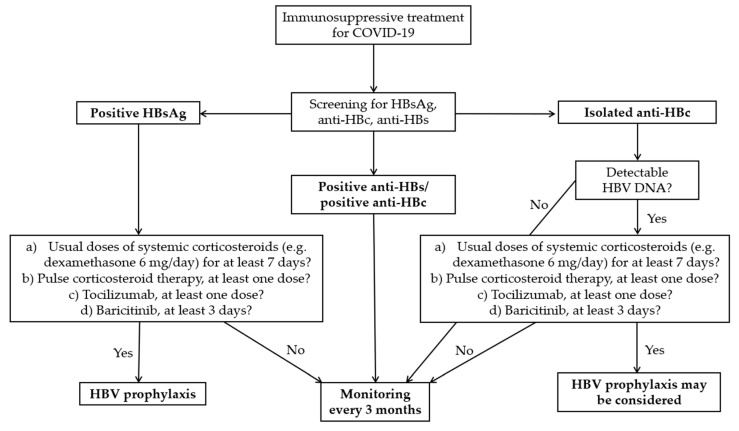
Proposed algorithm for management of COVID-19 patients receiving immunosuppressive treatment.

The data in Table 4, although comprising a small number of studies and patients, show a significant risk of HBVr in patients with SARS-CoV-2 infection receiving immunosuppressive treatment. It is reassuring that most cases of HBVr had no clinical implications. However, there were also case reports of clinically manifest reactivations, some resulting in patients’ death [20]. Most of these severe cases were in HBsAg-positive patients. Larger studies are needed in order to establish appropriate and cost-effective management for these patients. In the meantime, after analyzing the existing data, both from COVID-19 patients and from those with other diseases, we propose the algorithm illustrated in Figure 2.

First, we recommend performing an HBV triple screen panel that includes HBsAg, anti-HBs, and anti-HBc in all patients with SARS-CoV-2 infection for whom immunosuppressive treatment (e.g., tocilizumab, baricitinib, systemic corticosteroids) is indicated. It would be prudent to perform the same screening in patients receiving anakinra, given the limited data on the risk of HBVr related to this drug.

For HBsAg-positive patients, we suggest antiviral prophylaxis if they receive any of the following: (a) usual doses of systemic corticosteroids (e.g., dexamethasone, 6 mg/day) for at least 7 days; (b) pulse corticosteroid therapy, at least one dose; (c) tocilizumab, at least one dose; (d) baricitinib, for at least 3 days. We established these limits empirically and based on the observations made in our study, as well as in other studies performed to date. In addition, unlike other authors who encourage prophylaxis in all HBsAg-positive patients, we recommend a higher threshold, considering that there is also the possibility of HBVr after stopping HBV prophylaxis [38]. Obviously, patients who meet the criteria for HBV treatment even in the absence of immunosuppressants will receive antiviral treatment according to current guidelines. 

In patients with isolated anti-HBc, we suggest performing an HBV viral load test. If this is detectable and the criteria described in HBsAg-positive patients are met, HBV prophylaxis may be considered (Figure 2). However, given that reactivations are not clinically significant in these patients, monitoring may be sufficient. 

During the decision-making process, it is important to consider the possibility of subsequent monitoring of patients. For example, in this study, 11 of 44 patients (25%) were lost to follow-up, although they initially committed to come for re-evaluation. To these patients are added those who, from the start, refused enrolment in the study due to the need for a follow-up. However, the addressability of patients could be higher in other countries. Another criterion that could be considered is the degree of liver fibrosis. Existing data suggest that patients with more advanced liver fibrosis have higher morbidity and mortality associated with HBVr [13]. Therefore, we recommend that HBV prophylaxis be administered to these patients. Still, an impediment could be the long length of time until receiving the results of serological tests (such as FibroTest) or the unavailability of performing an elastography in a timely manner.

In anti-HBs-positive/anti-HBc-positive patients, we do not consider prophylaxis necessary, given the available data. Ideally, they should still be monitored after discharge.

Regarding anakinra treatment, there is no evidence related to the risk of HBVr in patients with COVID-19 or other diseases. However, data are limited, so we suggest screening for HBV and monitoring patients with positive markers for active or previous infection.

When HBV prophylaxis is decided upon, it is preferable to use nucleoside analogues with high genetic barrier (entecavir or tenofovir) for at least 6 months after the last dose of immunosuppressant [13]. However, the use of lamivudine is also supported if the viral load is below 2000 IU/mL, and it is anticipated that the prophylaxis will be of short duration [36]. If monitoring is decided, this ideally consists of repeating HBV DNA level, viral serological markers, and liver enzymes every 3 months. An alternative is to monitor liver enzymes and perform HBV viral load in case of increased aminotransferases [13,31].

### 4.6. Strengths and Limitations of the Study

This study is one of the few that offers an in-depth analysis of the risk of HBVr in patients co-infected with HBV and SARS-CoV-2 receiving immunosuppressive treatment. Moreover, the study had a prospective design, and the patients were monitored not only clinically and serologically but also virologically (by HBV DNA). Also, as far as we know, this is the only study that followed, virologically, patients diagnosed with COVID-19 during the Omicron variant circulation. This is important, given that its subvariants continue to be dominant worldwide. Furthermore, considering that SARS-CoV-2 could itself represent a trigger for HBVr, another strength of the study is that it also included a control group represented by COVID-19 patients who did not receive immunosuppressive treatment. Not least, this study also evaluated HBV seroprevalence in COVID-19 patients in order to clarify the importance of assessing the risk of HBVr in patients receiving immunosuppressants.

This study is limited by the small number of participants. However, compared to other studies, this study included a larger number of patients without protective factors for HBVr (antiviral treatment and/or positive anti-HBs). In addition, we analyzed, under a common denominator, all studies related to HBVr in COVID-19 patients receiving immunosuppressive treatment, allowing an overview of the results we have to date.

## 5. Conclusions

In conclusion, administration of immunosuppressants for COVID-19 treatment may result in a significant risk of HBVr in co-infected patients. We recommend performing an HBV triple screen panel that includes HBsAg, anti-HBs, and anti-HBc in all patients with SARS-CoV-2 infection for whom immunosuppressive treatment is indicated. We suggest HBV prophylaxis in HBsAg-positive patients receiving any of the following: (a) usual doses of systemic corticosteroids for at least 7 days; (b) pulse corticosteroid therapy, at least one dose; (c) tocilizumab, at least one dose; (d) baricitinib for at least 3 days. The fact that three of the four patients with HBVr in this study were diagnosed with COVID-19 during the Omicron variant circulation highlights that the risk assessment of HBVr in COVID-19 patients remains a current issue. Larger studies are needed in order to establish an appropriate and cost-effective management in these patients. Also, we emphasize the need for an explicit and uniform definition of HBVr.

## Figures and Tables

**Table 1:** Summary characteristics of study participants.

Variables	All Patients (N = 32)
Age (years), median (IQR)	67 (58.5–71.5)
Male, n (%)	19 (59.4%)
BMI (kg/m^2^), median (IQR)	28.5 (26.3–32.5)
Comorbidity, n (%)	
Hypertension	20 (62.5%)
Obesity	13 (40.6%)
Chronic pulmonary diseases	8 (25%)
Diabetes mellitus	7 (21.9%)
Alcohol use disorder	3 (9.4%)
Other comorbidities	4 (12.5%)
No comorbidities, n (%)	4 (12.5%)
COVID-19 severity, n (%)	
Mild	3 (9.4%)
Moderate	18 (56.3%)
Severe	9 (28.1%)
Critical	2 (6.3%)
COVID-19 diagnosis during the Omicron variant circulation, n (%)	18 (56.3%)
Immunosuppressive COVID-19 treatment administered during hospitalization, n (%)	
Systemic corticosteroids	23 (71.9%)
Anakinra	9 (28.1%)
Tocilizumab	4 (12.5%)
Baricitinib	4 (12.5%)
No immunosuppressive COVID-19 treatment, n (%)	9 (28.1%)
Hepatitis B virus status, n (%)	
HBsAg-positive	13 (40.6%)
HBsAg-negative/anti-HBc-positive	19 (59.4%)

Abbreviation: anti-HBc, hepatitis B core antibody; BMI, body mass index; HBsAg, hepatitis B surface antigen; IQR, interquartile range.

**Table 2:** Detailed characteristics of each study participant.

**(a) HBsAg-Positive Patients**						
**No.**	**Sex**	**Age** **(Years)**	**Treatment—Duration, Cumulative Dose**	**Viral Load at Baseline** **(IU/mL)**	**Viral Load at Follow-Up** **(IU/mL)**	**HBVr**
Patients who received tocilizumab, anakinra, and/or baricitinib						
1	F	58	ANK—7 days, 1000 mg DEX—12 days, 82 mg	164	362	No
2	M	43	TCZ—1 dose, 400 mg ANK—5 days, 1400 mg DEX—10 days, 128 mg	34	18	No
3	F	70	ANK—7 days, 1000 mg DEX—7 days, 72 mg	<10	<10	No
4	F	53	ANK—7 days, 1000 mg DEX—9 days, 68 mg	342	723	No
5 ^o^	M	61	ANK—4 days, 700 mg DEX—9 days, 76 mg	18	66	No
Patients who received systemic corticosteroids						
6	M	67	DEX—16 days, 146 mg	18	<10	No
7 ^o^	F	55	DEX—2 days, 16 mg	U	17	No
8 ^o^	F	56	DEX—7 days, 48 mg	179	17,378	Yes
Patients who did not receive any immunosuppressive treatment						
9	M	61	-	126	225	No
10 ^o^	M	49	-	646	1190	No
11 ^o^	F	34	-	77	<10	No
12 ^o^	F	77	-	11	118	No
13 ^o^	F	67	-	U	16	No
**(b) HBsAg-Negative/Anti-HBc-Positive Patients**						
**No.**	**Sex**	**Age** (**Years**)	**Treatment—Duration, Cumulative Dose**	**Viral Load at Baseline ^a^** **(IU/mL)**	**Viral Load at Follow-Up** **(IU/mL)**	**HBVr**
Patients who received tocilizumab, anakinra, and/or baricitinib						
14	M	75	TCZ—1 dose, 400 mg ANK—1 day, 100 mg DEX—15 days, 82 mg	U	<10	Yes
15	F	73	ANK—7 days, 1000 mg DEX—14 days, 152 mg	U	Positive anti-HBs ^b^	No
16	M	66	TCZ—1 dose, 400 mg ANK—6 days, 1200 mg DEX—33 days, 252 mg	U	U	No
17	M	56	ANK—1 day, 200 mg BARI—9 days, 36 mg DEX—9 days, 96 mg	-	U	No
18 ^o^	M	67	BARI—14 days, 56 mg DEX—9 days, 98 mg	-	U	No
19 ^o^	M	75	BARI—7 days, 28 mg DEX—7 days, 48 mg	-	U	No
20 ^o^	F	63	TCZ—1 dose, 800 mg BARI—3 days, 12 mg DEX—18 days, 140 mg	U	<10	Yes
Patients who received systemic corticosteroids						
21	F	59	DEX—5 days, 30 mg	-	U	No
22	M	68	DEX—5 days, 30 mg	-	U	No
23	M	62	DEX—19 days, 60 mg	-	U	No
24	M	83	DEX—12 days, 104 mg	-	U	No
25 ^o^	M	67	DEX—7 days, 38 mg	-	U	No
26 ^o^	M	62	DEX—5 days, 36 mg	-	U	No
27 ^o^	F	68	DEX—5 days, 30 mg	-	U	No
28 ^o^	M	80	DEX—9 days, 50 mg	U	10	Yes
Patients who did not receive any immunosuppressive treatment						
29 ^o^	M	75	-	-	U	No
30 ^o^	F	69	-	-	U	No
31 ^o^	M	74	-	-	U	No
32 ^o^	M	70	-	-	U	No

^a^ At the time of enrolment, serum samples were collected from all patients and stored at −20 °C. For patients in whom HBV DNA level was undetectable at follow-up, determination of baseline viral load was not considered necessary and was no longer performed. ^b^ Since the patient had positive hepatitis B surface antibody (anti-HBs) at follow-up, it was not considered necessary to perform the viral load. ^o^ Patients diagnosed with COVID-19 during the Omicron variant circulation. ANK, anakinra; BARI, baricitinib, DEX, dexamethasone; F, female; HBVr, hepatitis B virus reactivation; M, male; TCZ, tocilizumab; U, undetectable.

**Table 3:** Definitions of HBVr proposed by the medical associations dedicated to the management of liver diseases.

	HBVr in HBsAg-Positive Patients	HBVr in HBsAg-Negative/Anti-HBc-Positive Patients
KASL 2022 [12]	≥2 log (100-fold) increase in HBV DNA from baseline level	HBV DNA level > 2 log (100) IU/mL, if previously undetectable Reverse HBsAg seroconversion (reappearance of HBsAg)
APASL 2021 [13]	≥2 log (100-fold) increase in HBV DNA level, if previously detectable HBV DNA level > 2 log (100) IU/mL, if previously undetectable	Detectable HBV DNA, if previously undetectable Reverse HBsAg seroconversion (reappearance of HBsAg)
AASLD 2018 [14]	≥2 log (100-fold) increase in HBV DNA level, if previously detectable HBV DNA level ≥ 3 log (1000) IU/mL, if previously undetectable HBV DNA level ≥ 4 log (10,000) IU/mL, if previously unknown	Detectable HBV DNA * Reverse HBsAg seroconversion (reappearance of HBsAg)
AGA 2015 [11]	≥1 log (10-fold) increase in HBV DNA level, if previously detectable Detectable HBV DNA, if previously undetectable Reappearance of HBeAg	≥1 log (10-fold) increase in HBV DNA level, if previously detectable Detectable HBV DNA, if previously undetectable Reverse HBsAg seroconversion (reappearance of HBsAg)

* Without requiring a previously undetectable HBV DNA level. AASLD, American Association for the Study of Liver Diseases; AGA, American Gastroenterological Association; APASL, Asian Pacific Association for the Study of the Liver; KASL, Korean Association for the Study of the Liver. HBeAg, hepatitis B e-antigen.

**Table 4:** Number of patients with HBV–SARS-CoV-2 co-infection and immunosuppressive treatment who were followed virologically and experienced HBVr according to the definitions proposed by the medical associations dedicated to the management of liver diseases ^a^ (see Table 3 as well).

Medical Association	HBsAg-Positive Patients Who Experienced HBVr/ All Patients Followed, n/n	Study	HBsAg-Negative/ Anti-HBc-Positive Patients Who Experienced HBVr/ All Patients Followed, n/n	Study
KASL 2022 [12]	2/5	Liu et al. [4]	3/15	This work
	0/3	Camarero et al. [6]		
	1/8	This work		
Pooled rate of HBVr, %	18.75% (3/16)		20% (3/15)	
APASL 2021 [13]	2/5	Liu et al. [4]	0/15	This work
	0/3	Camarero et al. [6]		
	1/8	This work		
Pooled rate of HBVr, %	18.75% (3/16)		0%	
AASLD 2018 [14]	2/5	Liu et al. [4]	2/6	Tajez et al. [5] ^b^
	0/3	Camarero et al. [6]	3/15	This work
	1/8	This work		
Pooled rate of HBVr, %	18.75% (3/16)		23.8% (5/21)	
AGA 2015 [11]	2/5	Liu et al. [4]	3/15	This work
	1/3	Camarero et al. [6]		
	2/8	This work		
Pooled rate of HBVr, %	31.25% (5/16)		20% (3/15)	

^a^ Only patients without protective factors for HBVr (antiviral treatment and/or positive anti-HBs) were considered. ^b^ This study was considered only here, as the AASLD guideline is the only one that does not require a baseline viral load.

## Data Availability

The raw data supporting the conclusions of this article will be made available by the authors on request.

## Supplementary Materials

The following supporting information can be downloaded at https://www.mdpi.com/article/10.3390/jcm13206032/s1, Table S1. Biochemical parameters of study participants.
