# Supplementary material for: Risk of Hepatitis B Virus Reactivation in COVID-19 Patients Receiving Immunosuppressive Treatment: A Prospective Study
Source: J Clin Med. 2024 Oct 10;13(20):6032. doi: 10.3390/jcm13206032 (PMC11508539; doi:10.3390/jcm13206032)
Supplement: Supplementary file 1 [file jcm-13-06032-s001.zip › jcm-3233462-supplementary.pdf]

Table S1. Biochemical parameters of study participants

No.	ALT (x ULN)		AST (x ULN)		GGT (x ULN)		HBVr
	Peak during hospitalization	Follow-up	Peak during hospitalization	Follow-up	Peak during hospitalization	Follow-up	
HBsAg-positive patients							
1	**1.17**	**1.03**	**1.08**	0.94	0.8	0.45	No
2	**3.76**	0.78	**2**	0.54	**1.25**	0.42	No
3	**1.86**	0.66	0.65	0.83	**2.74**	0.6	No
4	0.8	0.54	1	0.64	0.95	0.37	No
5 °	**1.92**	0.54	**1.25**	0.56	**3.18**	0.62	No
6	0.7	0.4	0.4	0.5	0.72	0.45	No
7 °	1	0.34	0.97	0.69	0.53	0.46	No
8 °	0.91	0.8	**1.22**	0.92	**7**	**3.37**	Yes
9	**1.52**	0.4	0.93	**1.44**	**3.18**	**2.26**	No
10 °	**1.14**	0.66	0.69	0.4	**1.16**	0.89	No
11 °	0.6	0.46	0.8	0.61	0.42	0.44	No
12 °	0.37	0.34	0.72	0.72	0.35	0.39	No
13 °	0.91	0.71	**2.33**	0.61	**1.39**	**1.14**	No
HBsAg-negative/anti-HBc-positive patients							
14	**4.06**	0.38	**3.56**	0.4	0.78	0.29	Yes
15	0.83	0.43	**1.11**	0.8	0.86	0.42	No
16	**7.42**	0.4	**7**	0.54	**2.62**	0.56	No
17	**1.6**	**6.08**	**1.07**	**3.93**	**2.18**	**2.23**	No
18 °	**2.18**	0.54	**2.25**	0.58	**2.12**	0.46	No
19 °	0.5	0.4	0.47	0.39	N/A	0.33	No
20 °	**2.71**	0.6	**2.03**	0.64	**1.77**	0.44	Yes
21	0.63	0.88	0.86	0.94	0.44	0.37	No
22	0.54	0.22	0.57	0.29	0.25	0.22	No
23	**2.2**	0.8	**1.47**	**1.6**	**2.4**	**2.5**	No
24	**2.24**	0.34	0.85	0.42	**2.22**	0.48	No
25 °	**3.1**	**1.22**	0.98	0.64	**3.87**	**1.33**	No
26 °	0.5	0.32	0.56	0.52	0.48	0.23	No
27 °	**1.6**	**1.63**	**1.5**	**1.13**	**1.42**	0.95	No
28 °	0.86	0.26	0.54	0.29	0.75	0.33	Yes
29 °	0.48	0.42	0.5	0.49	0.5	0.42	No
30 °	0.17	0.11	**1.03**	0.78	0.37	0.32	No
31 °	0.2	0.24	0.47	0.42	0.46	0.49	No
32 °	0.36	0.4	0.63	0.39	0.37	0.29	No

° Patients diagnosed with COVID-19 during the Omicron variant circulation. ALT, alanine aminotransferase; anti-HBc, hepatitis B core antibody; AST, aspartate aminotransferase; GGT, gamma-glutamyl transferase; HBsAg, hepatitis B surface antigen; HBVr, hepatitis B virus reactivation; N/A, not available; ULN, upper limit of normal.
